# Deep Learning-Based Segmentation of Geographic Atrophy: A Multi-Center, Multi-Device Validation in a Real-World Clinical Cohort

**DOI:** 10.3390/diagnostics15202580

**Published:** 2025-10-13

**Authors:** Hasenin Al-khersan, Simrat K. Sodhi, Jessica A. Cao, Stanley M. Saju, Niveditha Pattathil, Avery W. Zhou, Netan Choudhry, Daniel B. Russakoff, Jonathan D. Oakley, David Boyer, Charles C. Wykoff

**Affiliations:** 1 Retina Consultants of Texas, Houston, TX 77070, USA; jessica.cao@retinaconsultantstexas.com (J.A.C.); stanley.saju@gmail.com (S.M.S.); avery.zhou@retinaconsultantstexas.com (A.W.Z.); ccwmd@retinaconsultantstexas.com (C.C.W.); 2 Clinical School of Medicine, University of Cambridge, Cambridge CB2 0SP, UK; guneet.s.sodhi@gmail.com; 3 Vitreous Retina Macula Specialists of Toronto, Toronto, ON M8X 2X3, Canada; pattathiln@gmail.com (N.P.); netan.choudhry@vrmto.com (N.C.); 4 Department of Ophthalmology and Vision Sciences, University of Toronto, Toronto, ON M5T 3A9, Canada; 5 Voxeleron Inc., Austin, TX 78750, USA; daniel@voxeleron.com (D.B.R.); jonathan@voxeleron.com (J.D.O.); 6 Retina-Vitreous Associates Medical Group, Los Angeles, CA 90017, USA; retinaldoc@aol.com; 7 Keck School of Medicine, University of Southern California, Los Angeles, CA 90033, USA

**Keywords:** artificial intelligence, age-related macular degeneration, deep learning, geographic atrophy, retina

## Abstract

**Background:** To report a deep learning-based algorithm for automated segmentation of geographic atrophy (GA) among patients with age-related macular degeneration (AMD). **Methods:** Validation of a deep learning algorithm was performed using optical coherence tomography (OCT) images from patients in routine clinical care diagnosed with GA, with and without concurrent nAMD. For model construction, a 3D U-Net architecture was used with the output modified to generate a 2D mask. Accuracy of the model was assessed relative to the manual labeling of GA with the Dice similarity coefficient (DSC) and correlation r^2^ scores. **Results:** The OCT data set included 367 scans from the Spectralis (Heidelberg, Germany) from 55 eyes in 33 subjects; 267 (73%) scans had concurrent nAMD. In parallel, 348 scans were collected using the Cirrus (Zeiss), from 348 eyes in 326 subjects; 101 (29%) scans had concurrent nAMD. For Spectralis data, the mean DSC score was 0.83 and r^2^ was 0.91. For Cirrus data, the mean DSC score was 0.82 and r^2^ was 0.88. **Conclusions:** The reported deep learning algorithm demonstrated strong agreement with manual grading of GA secondary to AMD on the OCT data set from routine clinical practice. The model performed well across two OCT devices as well as amongst patients with GA with concurrent nAMD, suggesting applicability in the clinical space.

## 1. Introduction

Geographic atrophy (GA) is an advanced stage manifestation of non-exudative age-related macular degeneration (AMD), representing one of the leading causes of vision loss in adults over 50 years of age [1]. GA is characterized by the progressive degeneration of the choriocapillaris, retinal pigment epithelium (RPE), and overlying photoreceptors, resulting in irreversible central vision loss [2].

Recently, two intravitreal anti-complement therapies, pegcetacoplan and avacincaptad pegol, were introduced in the United States for the treatment of GA [3,4]. These therapies were approved by the Food and Drug Administration based on structural endpoints. Both therapies demonstrated a slower rate of progression of GA relative to sham-treated populations, although neither demonstrated any prespecified visual benefit. Given the introduction of these therapies, the accurate measurement of GA area and its progression is of heightened importance to support the clinical management of patients being considered for or receiving pharmacotherapy in routine clinical practice.

GA is typically diagnosed using non-invasive imaging techniques including optical coherence tomography (OCT), fundus autofluorescence (FAF), and fundus photography [5,6]. OCT is particularly useful for identifying GA due to its widespread availability in clinical practice, ability to identify subtle morphological changes in early disease, and ease of capture. In 2018, OCT was proposed as the gold standard to diagnose GA by the international expert Consensus Definition for Atrophy Associated with Age-Related Macular Degeneration on OCT group [7]. Specific OCT criteria to diagnose complete RPE and outer retinal atrophy (cRORA) were proposed. While these criteria may accurately define GA, they are challenging for ophthalmologists to methodically apply across thousands of B-scans in routine clinical practice. Toward this end, artificial intelligence (AI) can serve to simplify the process by automating the identification of GA [8,9].

Different approaches to segmenting GA in image data have been developed by device manufacturers and research groups. For OCT, the only software with clinical approval is the advanced retinal pigment epithelial (RPE) analysis tool (ARPET; Carl Zeiss Meditec, Dublin, CA, USA). The method proceeds by segmenting 3D OCT data and using the segmentation to create a 2D en face slab image based only on image data extending from 65 μm to 400 μm below a fit to the RPE [10]. Relying on an accurate layer segmentation and performing the segmentation only in 2D are detrimental to this approach’s performance.

The advent of deep learning has resulted in several fully automated applications. Methods based on segmenting each B-scan individually using a 2D U-Net architecture and compiling these results across the slices has been previously reported [11,12]. Researchers have overcome the limitations of the aforementioned approaches by working directly with the 3D data based on a novel architecture that modifies a U-Net to encode in 3D and decode in 2D [13]. In this approach, the skip connections, common to the U-Net architecture, drop one dimension from the data and go from 3D to 2D. Our extension to that method adds residual blocks to allow for better feature representations [14]. In the following, we examine the ability of this 3D U-Net-type architecture to identify GA on OCT data from real-world patient data.

## 2. Methods

### 2.1. Data Collection and Grading

Data was collected from two sites retrospectively, the Retina Consultants of Texas (RCTX, Houston, TX, USA) and Retina-Vitreous Associates (RVA, Los Angeles, CA, USA), from 2016 and 2022. Inclusion criteria were a clinical diagnosis of GA with accompanying OCT imaging; eyes with concurrent nAMD were also included. Institutional review board approval was obtained from Advarra. The present study was conducted in accordance with the Declaration of Helsinki.

Optical coherence tomography imaging was performed on the Heidelberg Spectralis (Heidelberg Engineering, Heidelberg, Germany) at RCTX and the Zeiss Cirrus (Carl Zeiss Meditec, Dublin, CA, USA) at RVA as part of routine clinical care. Both used similar protocols for capturing data at the macula using a 20 degree (~6 mm^2^) lateral field of view (FOV).

For Spectralis, the macular scan protocol was used with an ART setting of up to 10 averaged frames. The volumes comprised 49 slices, where each B-scan was 512 by 496 pixels. The Spectralis data included near-infrared (nIR) images, which were across a 30-degree FOV and used 100-frame averaging. A quality index of at least 25 was required for inclusion; this guidance came from the manufacturer.

For Cirrus data, the macular protocol included 200 B-scans, each with 200 by 1024 pixels. The Zeiss macular protocol does not use frame averaging. A minimal signal score of five was required for inclusion. No associated nIR data was collected by the device.

### 2.2. Grading

**Figure 1:**
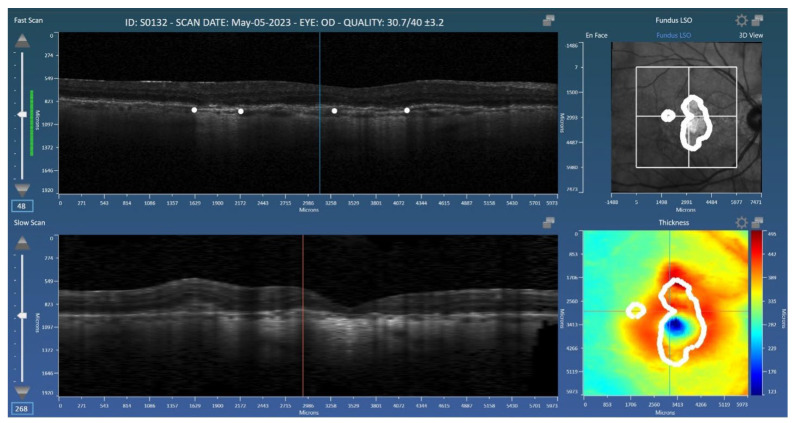
Representation of the Orion application used for the grading of geographic atrophy (GA). Graders placed landmarks (white dots) at the boundaries of GA on the B-scans. The resulting 2D mask (GA area) is shown in the *en face* view (**top right**).

Manual grading was performed by two expert graders who delineated areas of cRORA on OCT based on the Classification of Atrophy Meetings (CAM) criteria as previously described [7]. Graders marked areas of cRORA using the Orion (Voxeleron Inc., Austin, TX, USA) application [15]. Specifically, graders marked the bounds of atrophy on the B-scan cross-sections (Figure 1). The delineated areas are subsequently represented two-dimensionally on the OCT en face view for confirmation of the correct demarcation. Graders were masked to the nIR images collected in the Spectralis data set for consistency with the Cirrus labeling, which had no nIR data. As the scans had already been filtered based on quality, all volumes were gradable. No repeat grading was performed.

### 2.3. Deep Learning Architecture

**Figure 2:**
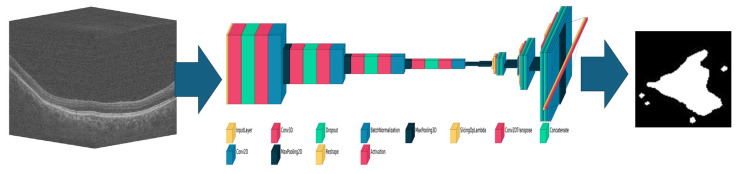
Schematic of the convolutional neural network-based 3D to 2D network used for geographic atrophy segmentation. Once trained, an input 3D optical coherence tomography volume (**left**) will generate an output 2D mask (**right**) based on the learned weights and parameters of the network (**middle**).

A 3D U-Net architecture was used for deep learning with the output modified to generate a 2D mask (Figure 2). It extends the approach of Lachinov by adding attention blocks to the architecture in an effort to better localize the region in which the atrophy is imaged [13]. Explicit localization has previously been implemented using layer segmentation, but here we do this in 3D and in a single architecture with the localization learned [10,11].

To alleviate memory management issues caused by large 3D volumes, for the Spectralis data, the volume was sub-sampled to 128-by-128-by-64 pixels (i.e., to 64 B-scans each of 128^2^ pixels). The Cirrus data, due to the more isotropic distribution, was sub-sampled instead to 128-by-128-by-128 pixels (i.e., to 128 B-scans each of 128^2^ pixels). The output was a 2D mask of GA at a resolution of 128-by-64 pixels and 128-by-128 pixels for Spectralis and Cirrus, respectively. For the Spectralis implementation, the architecture was also capable of receiving the 2D nIR images in a second input channel to see if this additional information would improve the algorithm’s performance.

Training each of the models used 450 epochs with a batch size of 32. A patience parameter was set to 70 meaning that, if after 70 iterations no improvement was seen in the performance relative to the validation split (a part of the training set), the learning stopped. The AdamW optimizer was used with an initial learning rate of 5 × 10^−5^. The loss function weighed both the cross entropy and the DICE coefficient. All training was run on a Dell Precision 7920 Workstation (Dell Technologies, Round Rock, TX, USA), housing two NVIDIA A6000s connected via the NVLINK system (NVIDIA Corportation, Santa Clara, CA, USA). On average, each training fold took 90 min using both GPUs using distributed learning. The implementation used TensorFlow version 2.15.

### 2.4. Analysis Methods

N-fold cross-validation was performed. In this scenario, data is split N times, where at each of the N-folds, 1/Nth of the entire data set is set aside for testing and the remaining for training. Training further splits the data into train and validate groups, where the latter is used to determine when the training iterations stop. For the present study, five folds were used. For repeat cases from the same subject, the folds were structured such that at no time was data from a single subject represented both in the train and test sets. Dice Similarity Scores (DSC) and correlation (r^2^) between manual and automated areas of atrophy were calculated. Student *t*-test was used to compare scores in the GA-only and GA-with-nAMD data subsets. A *p*-value of <0.05 was considered statistically significant. All final analyses were performed using Matlab and its Statistics Toolbox (Mathworks, Natick, MA, USA).

## 3. Results

For the Spectralis data, 367 scans were used from 55 subject eyes in 33 subjects. The data was collected over a period of eight years. All 367 scans had clinically diagnosed GA, which was confirmed during the grading process. Of the 367 scans, 267 (73%) had been clinically diagnosed with concurrent nAMD, while the remaining 100 (27%) did not have concurrent nAMD.

The Cirrus data subset comprised 348 subject eyes from 326 subjects representing 348 total scans. All clinically diagnosed cases of GA were confirmed during the grading process. Of the 348 scans, 101 (29%) had presumed nAMD, while 247 (70.1%) did not have concurrent nAMD.

For the Spectralis data, the mean DSC score was 0.83 and r^2^ was 0.91 (Table 1). With the addition of nIR data, there was no significant change in the mean DSC score (0.83) and r^2^ (0.91). For the Cirrus data, the mean DSC score was 0.82 and r^2^ was 0.88.

Stratification of the Spectralis data based on the presence of concurrent nAMD did not demonstrate a significant difference in the mean DSC score. However, there was a statistically significant decrease in correlation scores when comparing eyes without (0.97) with those with concurrent nAMD (0.85, *p* < 0.05). For the Cirrus data, there was no significant difference in the DSC scores or correlations when stratifying based on the presence of nAMD.

**Figure 3:**
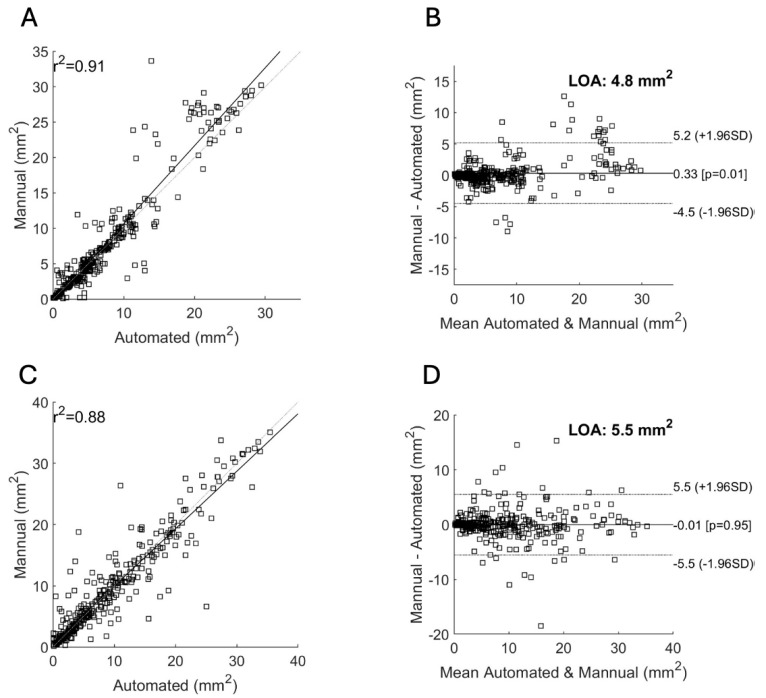
Correlation of area measurements for the Spectralis (**A**,**B**) and Cirrus (**C**,**D**) data sets. (**A**,**C**): Scatter plot showing the manual area measurements (*y*-axis) versus the automated measurements (*x*-axis) and their resulting correlation for the Spectralis (**A**) and Cirrus (**C**) data sets. (**B**,**D**): Bland–Altman plots showing the limits of agreement (LOA) between automated and manual measurements for the Spectralis (**B**) and Cirrus (**D**) data sets. The *y*-axis shows the difference between the manual and automated measurements and the *x*-axis their average.

**Figure 4:**
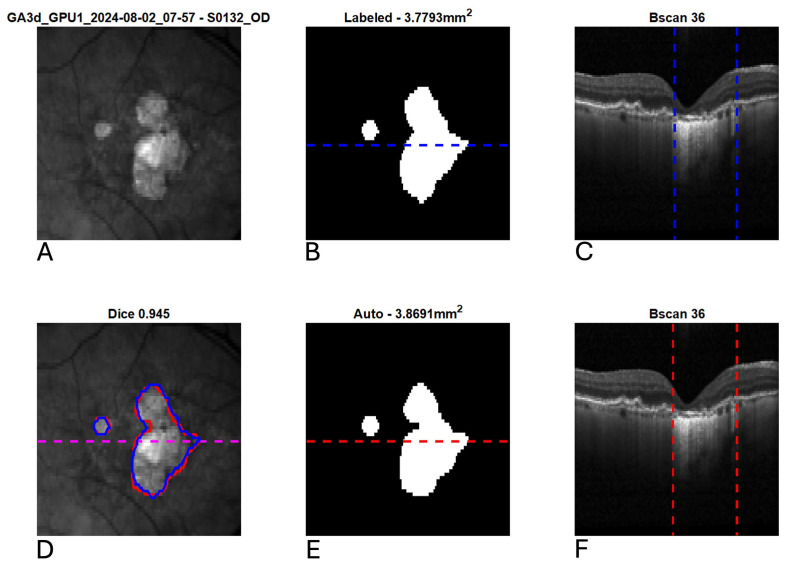
Example segmentation of geographic atrophy (GA) from optical coherence tomography (OCT). Blue indicates manual delineation; red indicates automated segmentation. (**A**): Input near-infrared en face image. (**B**,**E**): 2D GA segmentation maps from manual and automated delineation, respectively. (**C**,**F**): Representative B-scans (through horizontal lines in (**B**,**E**)) with GA delineated (vertical lines). (**D**): En face near-infrared with manual (blue) and automated (red) segmentations of GA overlain.

**Figure 5:**
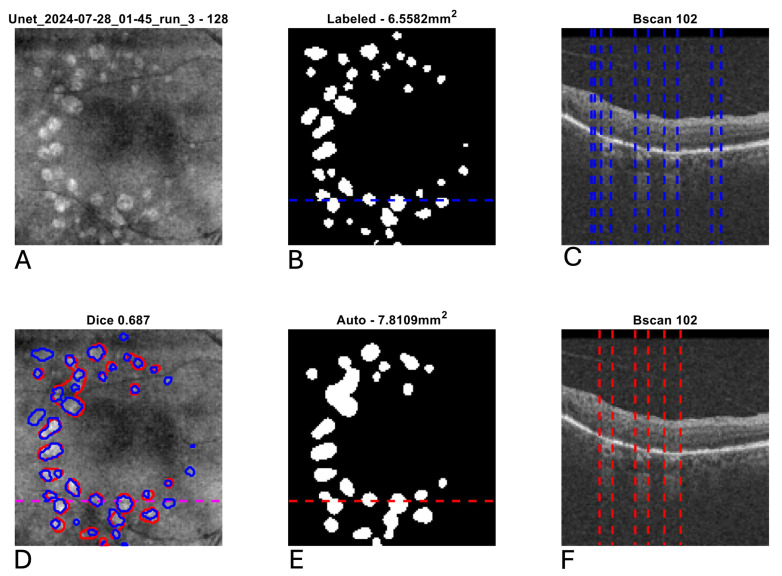
Example segmentation of multifocal geographic atrophy (GA) from optical coherence tomography (OCT). Blue indicates manual delineation; red indicates automated segmentation. (**A**): Input near-infrared en face image. (**B**,**E**): 2D GA segmentation maps from manual and automated delineation, respectively, demonstrating a tendency for the automated segmentation to coalesce smaller GA lesions relative to the manual grading. (**C**,**F**): Representative B-scans (through horizontal lines in (**B**,**E**)) with GA delineated (vertical lines). (**D**): En face near-infrared with manual (blue) and automated (red) segmentations of GA overlain.

**Figure 6:**
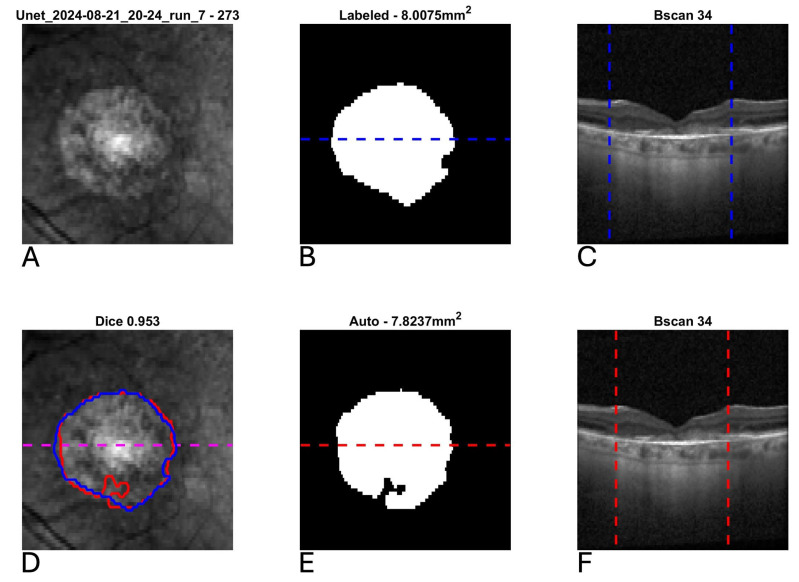
Example segmentation of geographic atrophy (GA) from optical coherence tomography (OCT) in a patient with concurrent neovascular age-related macular degeneration and subretinal fibrosis. Blue indicates manual delineation; red indicates automated segmentation. (**A**): Input near-infrared en face image. (**B**,**E**): 2D GA segmentation maps from manual and automated delineation, respectively, demonstrating high agreement. (**C**,**F**): Representative B-scans (through horizontal lines in (**B**,**E**)) demonstrating subretinal fibrosis with GA delineated (vertical lines). (**D**): En face near-infrared with manual (blue) and automated (red) segmentations of GA overlain.

**Figure 7:**
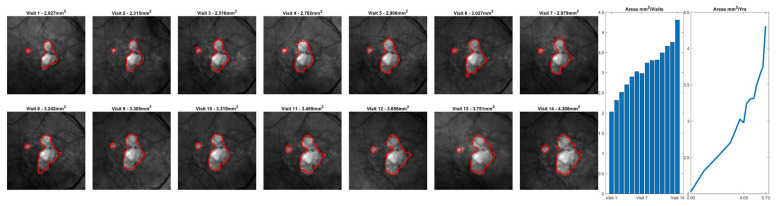
Representative longitudinal analysis of a fixed geographic atrophy (GA) segmentation model applied to data from a single patient over 14 visits. While this data was not used as part of the reported analysis, it demonstrates the intended use case for the reported GA algorithm. The plots on the right side of the image demonstrate the growth of GA (in mm^2^) over each visit.

Figure 3 shows the scatter plot and Bland–Altman plot for automated and manual measurements for the Spectralis and Cirrus data sets. Supplementary Figure S1 shows the same plots using OCT and nIR data together in the Spectralis data set. Figure 4, Figure 5 and Figure 6 show example segmentations in cases of GA, multifocal GA with many loci, and a case of concurrent atrophy and neovascular AMD. Finally, Figure 7 shows an example longitudinal analysis of how the tool may be employed in the clinical setting.

## 4. Discussion

The current study demonstrates the ability of a deep learning algorithm to segment GA using OCT from a real-world clinical cohort using two different hardware OCT devices. Unlike many prior studies, which excluded patients with nAMD and many of which are based on clinical trial data, the present investigation included data from patients with GA secondary to AMD both with and without concurrent nAMD in routine clinical practice settings [16,17,18,19,20]. Overall, the algorithm demonstrated strong agreement with manual grading as reflected by mean Dice scores and correlation > 0.8 across devices and disease states.

Validation studies of GA segmentation algorithms using patient cohorts with concurrent nAMD are rare [21]. Yet patients with GA are at relatively high risk of developing concurrent exudative disease. In a study of more than 23,000 patients with GA and no exudative disease at baseline, 25% of eyes developed nAMD during at least 3 years of follow up [22]. Furthermore, clinical trials leading to the approval of complement inhibitors for GA and subsequent post-approval studies have demonstrated higher risk of choroidal neovascular membrane formation with treatment [23,24]. Therefore, in order to be more broadly clinically useful, deep learning algorithms for automated GA segmentation should be able to perform well among patients without and with concurrent neovascular disease.

In the current study, the results are encouraging with respect to the performance of the algorithm for GA in the presence of concurrent nAMD, with DSC and correlation scores > 0.8 for both the Cirrus and Spectralis data. Comparatively, one prior study assessing atrophy among patients with nAMD reported a DSC score of 0.706 [21]. Another study by Liefers investigated the ability of a deep learning algorithm to detect multiple OCT features including RPE loss in nAMD [25]. The reported DSC score for the detection of RPE loss in that study was 0.471. The results of the present study represent the highest DSC and correlation scores reported for the automated detection of GA in patients with nAMD by a deep learning algorithm to date.

In the current sub-analysis comparing patients with and without concurrent nAMD, the DSC and correlation scores did not significantly differ within the Cirrus data. For the Spectralis data, however, while the DSC scores did not significantly differ with and without concurrent nAMD, the correlation scores decreased significantly in the presence of nAMD. This suggests that the AI’s correct identification of GA is not diminished in the presence of neovascular AMD but that the AI is less accurate in estimating the total size of the GA. The reasons driving this difference and its presence only in the Spectralis data subset warrants further study. While fluid on OCT reflects very little light, nAMD can lead to features like subretinal fibrosis that may be more difficult to distinguish from GA on OCT [26]. Additionally, updated analysis with higher-density line scans would be useful to determine if greater OCT resolution would lead to higher scores.

Importantly, data was derived from two widely used OCT devices from two different manufacturers. Device agnostic algorithms are more likely to have broad clinical utility, given the usage of different devices in retina clinics globally [27]. Another strength of the current work is the incorporation of real-world data from retina clinics rather than use of data derived from a clinical trial. A challenge with many AI-based algorithms is their generalizability outside of the curated data on which they are trained [28]. Clinical trials for GA often have robust exclusion criteria based on the size and morphology of the GA lesion, presence of nAMD, prior intravitreal therapy, and other factors that may make results less generalizable [29]. The present study is likely to be more generalizable given the inclusion of data from two devices from two independent retina clinics with different patient populations. Relative to ground truth assessment, we have shown excellent correlation for both devices along with narrow limits of agreement and very high DSC scores.

GA area, as measured using imaging, is relevant and clinically meaningful, and its progression, in general, is more predictable than our current ability to measure change in progressive visual dysfunction among affected patients. The FDA has designated the area of GA as an approvable-endpoint in the development of treatments for GA [30]. To data, FDA-approved pharmacotherapies for GA have utilized FAF as the primary tool to assess GA lesion growth over time. FAF relies on the detection of natural fluorescence emitted by lipofuscin, a pigment that accumulates in the RPE and has several factors limiting its routine, longitudinal clinical use. FAF imaging is two-dimensional and offers no cross-sectional information. Furthermore, FAF imaging is time-intensive and unpleasant for patients. In comparison, OCT imaging is rapid, extremely well tolerated by patients, and universally utilized in retina clinics. Furthermore, OCT allows detailed and quantitative assessment of individual retinal layers including the ellipsoid zone (EZ) [31]. The importance of OCT in the assessment of GA is reflected both in the acceptance of OCT-based metrics as a new FDA-approvable endpoint and in the definition of GA published by the CAM group, which uses OCT rather than FAF [7,32].

While OCT is a critical tool in the assessment of GA, grading GA based on OCT is exceptionally time-intensive and completely impractical at the volume needed within the flow of a routine retina clinic. The current work represents a practical approach for achieving this in an automated fashion, considering how well the automated approach compared with expertly labeled data. Outside of use in clinical practice, the automated and quantitative assessment of GA lesions by OCT will also be useful for applications such as consideration of patients for ongoing clinical trials; such automated methods offer the opportunity for more efficient and accelerated clinical trial recruitment.

There are limitations that must be considered in interpreting these results. Unlike clinical trial data, which is independently reviewed by a reading center, diagnosis of GA and nAMD in the current data set were based on physician clinical judgment. Additionally, while the Spectralis volumes utilized in the current work had 49-line scans, the algorithm may have demonstrated better performance if a higher-density scan pattern had been utilized such as 97-line scans. Furthermore, the Spectralis data was collected from a smaller number of patients followed longitudinally compared to the Cirrus data, which was more cross-sectional in nature. Finally, Spectralis data with a noise score below the manufacturers’ recommendations were excluded from this study; data with increased noise causes a drop in algorithm performance and results in data that is harder to grade. It is an open question as to whether or not such data should be included in the training data given, and one that is not addressed in this study.

## 5. Conclusions

In summary, the reported deep learning-based algorithm demonstrated excellent performance relative to manual grading of GA in patients with AMD, with correlations and mean DSC values of >0.8 across both devices. The data used to train and test the model is representative of real-world clinical data, consisting of eyes with and without concurrent nAMD and images from two devices.

With the advent of therapeutics designed to slow the progression of GA, such automated analysis tools will have wide applicability in clinical management, trial recruitment, and endpoint analysis. Future work is needed to validate these automated analysis tools to ensure that they perform well across a diverse set of patients and imaging platforms.

## Figures and Tables

**Table 1:** Geographic atrophy automated segmentation algorithm performance across devices.

	Cirrus		Spectralis OCT Only		Spectralis OCT + nIR	
	GA only	GA + Treatment	GA only	GA + nAMD	GA only	GA + nAMD
	*N* = 247	*N* = 101	*N* = 100	*N* = 267	*N* = 100	*N* = 267
**Average DSC**	0.82		0.83		0.83	
	0.82	0.84	0.80	0.83	0.82	0.83
***p*-Values**	0.18		0.08		0.77	
**Correlation (r^2^)**	0.88		0.91		0.91	
	0.89	0.87	0.97	0.85	0.98	0.84
*p*-Values *	0.26		*p* < 0.05		*p* < 0.05	

* *p*-values are for comparisons between geographic atrophy (GA) only data and GA with concurrent neovascular AMD.

## Data Availability

The data presented in this study are available on request from the corresponding author due to clinical patient information.

## Supplementary Materials

The following supporting information can be downloaded at https://www.mdpi.com/article/10.3390/diagnostics15202580/s1, Figure S1: Correlation of area measurements for the Spectralis OCT data set with inclusion of near-infrared images as input to the neural network. Left: scatter plot showing the manual area measurements (y-axis) versus the automated measurements (x-axis) and their resulting correlation (r^2^ = 0.91). Right: Bland-Altman plot showing the limits of agreement (LOA) between automated and manual measurements. The y-axis shows the difference between the manual and automated measurements and the x-axis their average. The correlation is the same, but the limits of agreement are slightly narrower. Table 1 summarizes overall findings.

## Abbreviations

GA	geographic atrophy
OCT	optical coherence tomography
AMD	age-related macular degeneration
DSC	Dice similarity coefficient
FAF	Fundus autofluorescence
AI	artificial intelligence
RPE	retinal pigment epithelium
RCTX	retina consultants of Texas
RVA	retina-vitreous associates
ART	automatic real-time tracking
cRORA	complete RPE and outer retinal atrophy
CAM	classification of atrophy meetings
GPU	graphics processing unit
LOA	limits of agreement
FDA	Food and Drug Administration
EZ	ellipsoid zone
