# Supplementary figures and images for: Deep Learning-Based Segmentation of Geographic Atrophy: A Multi-Center, Multi-Device Validation in a Real-World Clinical Cohort
Source: Diagnostics (Basel). 2025 Oct 13;15(20):2580. doi: 10.3390/diagnostics15202580 (PMC12562695; doi:10.3390/diagnostics15202580)

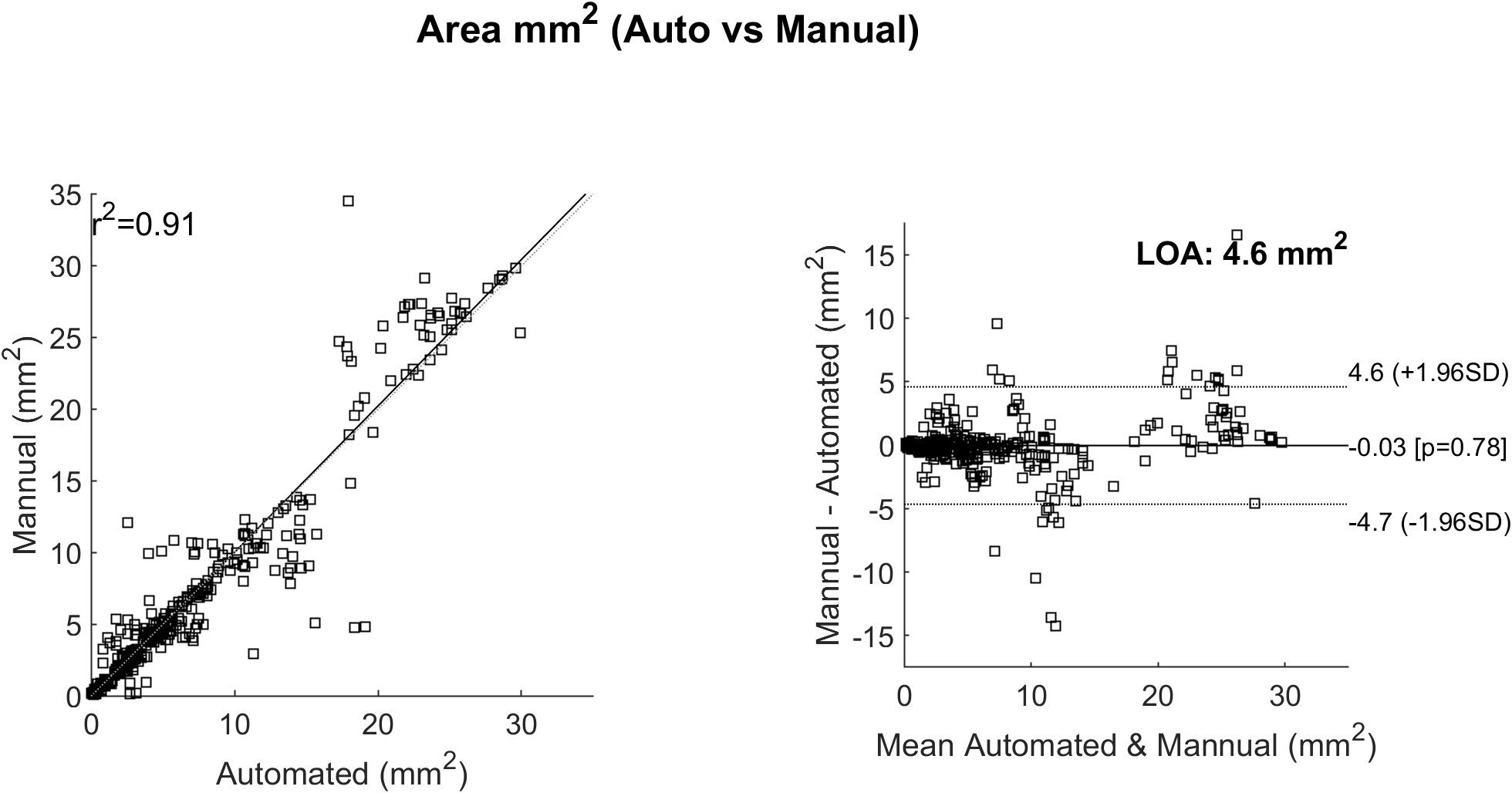

Supplement: Supplementary file 1 [file diagnostics-15-02580-s001.zip › Figure S1.jpg]
